# Supplementary material for: An innate contribution of human nicotinic receptor polymorphisms to COPD-like lesions
Source: Nat Commun. 2021 Nov 4;12:6384. doi: 10.1038/s41467-021-26637-6 (PMC8568944; doi:10.1038/s41467-021-26637-6)
Supplement: Supplementary file 1 — Supplementary Information [file 41467_2021_26637_MOESM1_ESM.pdf]

An innate contribution of human nicotinic receptor polymorphisms to Chronic Obstructive Pulmonary Disease-like lesions

Authors: Julie Routhier[1], Stéphanie Pons[2], Mohamed Lamine Freidja[1,3], Véronique Dalstein[1,4], Jérôme Cutrona[1], Antoine Jonquet[1], Nathalie Lalun[1], Jean-Claude Mérol[1,5], Mark Lathrop[6], Jerry A. Stitzel[7], Gwenola Kervoaze[8], Muriel Pichavant[8], Philippe Gosset[8], Jean-Marie Tournier[1], Philippe Birembaut[1,4,9], Valérian Dormoy[1,9], Uwe Maskos[2,9]

Supplemental Data

Variable	Epithelial phenotype								
	Inflammation			Secretory cell hyperplasia			Normal epithelium		
	0-1<br>n (%)	2-3<br>n (%)	p	< 10%<br>n (%)	≥ 10%<br>n (%)	p	< 50%<br>n (%)	≥ 50%<br>n (%)	p
Age			0.0394			0.7102			0.7334
Mean ± SD	53.8±13.4 (50)	48.1±16.8 (50)		51.8±14.7 (30)	50.6±15.7 (70)		50.7±13.8 (72)	51.7±19 (28)	
Allergy/ Asthma			0.123			0.261			0.086
Yes (n=57)	33 (27)	24 (19)		20 (16)	37 (30)		37 (30)	20 (16)	
No (n=66)	29 (24)	37 (30)		17 (14)	49 (40)		52 (42)	14 (12)	
Genotype			0.003			<0.0001			0.014
WT (G/G) (n=39)	28 (23)	11 (9)		22 (18)	17 (14)		22 (18)	17 (14)	
HT (G/A) (n=59)	26 (21)	33 (27)		12 (10)	47 (38)		45 (36)	14 (12)	
HO (A/A) (n=25)	8 (6)	17 (14)		3 (2)	22 (18)		22 (18)	3 (2)	
Gender			0.417			0.529			0.762
Male (n=75)	40 (33)	35 (28)		20 (16)	37 (30)		55 (45)	20 (16)	
Female (n=48)	22 (18)	26 (21)		17 (14)	49 (40)		34 (27)	14 (12)	

Supplementary Table 1: Univariate analysis of the relationship between *CHRNA5* genotype and epithelial remodelling in human nasal polyps

Target	Manufacturer	Catalog Nb	Clone	Lot Number	Dilution
Flow cytometry					
FITC-I-Ab	Miltenyi Biotech	130-102-168	M5/114.15.2	5161107010	1/200
PE-F4/80	Miltenyi Biotech	130-102-422	REA126	5151203251	1/200
PerCP Cy5.5 CD103	BD Biosciences	563637	M290	7104716	1/300
PE-Cy7-CD11c	BD Biosciences	558079	HL3	8083701	1/500
APC-CCR2	R&D systems	FAB5538A	475301	ABLE0314121	1/200
AF700-CD86	BD Biosciences	560581	GL1	7034805	1/100
APC-H7-Ly6G	BD Biosciences	560600	1A8	6263521	1/500
V450-CD11b	BD Biosciences	560455	M1/70	5322624	1/300
V500-CD45	Miltenyi Biotech	130-102-412	30F11	5150423227	1/300
BV605-Ly6C	Biolegend	128036	HK1.4	B245767	1/300
BV786-CD64	BD Biosciences	741024	X54-5/7.1	7319699	1/500
PE-CF594-SiglecF	BD Biosciences	562757	E50-2440	7128860	1/300
FITC-CD5	Miltenyi Biotech	130-102-574	53.7.3	5141118262	1/200
Tetramer mCD1d 167ms	NIH facility	30663		2016-06-26	1/500
PerCP Cy5.5 NK1.1	Miltenyi Biotech	130-103-963	PK136	5141202377	1/300
PE-Cy7 CD4	Miltenyi Biotech	130-102-411	GK1.5	5150601111	1/500
APC-CD25	Miltenyi Biotech	130-102-550	PC61	5141202369	1/200
AF700-CD69	BD Biosciences	561238	H12F3	7054759	1/500
APC-Vio770 TCRγδ	Miltenyi Biotech	130-104-016	GL3	5171122319	1/100
V450-TCRb	Miltenyi Biotech	130-104-815	REA318	5171122321	1/100
V500-CD8	BD Biosciences	130-109-252	REA601	5161107165	1/500
BV605CD45	Biolegend	103140	30F11	B235438	1/300
FITC IgG1	BD Biosciences	555749	MOPC21	5225509	1/100
IgG- isotype control	Abcam	ab37415			18μg/mL
NGFR	Abcam	ab8875			18 μg/ml
GFP	Invitrogen	A-6455			1/750
Goat anti-rabbit AF488	Abcam	ab150077			1/1000
Goat anti-rabbit AF633	Thermo Fisher Sc	A-21070			1/1000
Immunohistochemistry					
CC10	Abcam	ab40873		GR21852011	1/3200
Acetylated tubulin	Sigma-Aldrich	T6793	6-11B-1	034M4828	1/1000
GFP	Thermo Fisher Sc	A-6455		1826342	1/1000
Adenylate cyclase-1	Santa Cruz	Sc-25743		L1208	1 μg/ml
Adenylate cyclase-3	Santa Cruz	Sc-32114		B1413	1 μg/ml
Adenylate cyclase-8	Santa Cruz	Sc-20764		C1811	1 μg/ml
CK5	Abcam	ab53121			5μg/ml
Pan-cytokeratin	Elabscience	E-AB-33599		DK9774	10 μg/ml
CD45	Dako	M0701	2B11+PD7/26		1/50
CD68-KP1	Dako	M0814	KP1		1/400
NGFR	Abcam	ab8875			10 μg/ml
IgG- isotype control	Abcam	ab37415			10μg/mL
Goat anti-rabbit AF488	Molecular Probes	A11008		1885240	2 μg/ml
Donkey anti-rabbit AF488	Molecular Probes	A21206		1796375	2 μg/ml
Goat anti-rabbit AF594	Molecular Probes	A11012		1084427	2 μg/ml
Donkey anti-goat AF594	Molecular Probes	A11058		1736986	2 μg/ml
Immunoblotting					
Phospho-p65	Cell SIgnaling	#3031			1/1000
p65	Santa-Cruz	Sc-372			1/1000
Actin	Sigma-Aldrich	A2066			1/1000

Supplementary Table 2: Antibody panels for antigen presenting cells (APC) and lymphocyte labelling for flow cytometry, immunohistochemistry, and immunoblotting

Gene	Forward	Reverse
CHRNA5	5’-CGCCTTTGGTCCGCAAGATA-3’	5’-TGCTGATGGGGGAAGTGGAG-3’
Hprt	5'-ACATTGTGGCCCTCTGTGT-3'	5'-TGTAATCCAGCAGGTCAGCA-3'
NF-κBiα	5'-GAATTGCTGAGGCACTTCTGAA-3'	5'-GGGGTATTTCCTCGAAAGTCTC-3'
Tgm-1	5'-CCCCAGACCTTTCTCTTACGTTAC-3'	5'-CTCCACATTCCTGACCAACA-3'
Trp63	5'-TTTTGAAACTTCACGGTGTGC-3'	5'-GAAACGCTGGATGTAAGGGTC-3'
Tnf-α	5'-AGCACAGAAAGCATGATCCG-3'	5'-ACCCCGAAGTTCAGTAGACAG-3'
Il-1β	5'-CCTGTGTTTTCCTCCTTGCCT-3'	5'-TCTCAGCTTCAATGAAAGACCTC-3'
Il-6	5'-GAGACTTCCATCCAGTTGCC-3'	5'-AAGTAGGGAAGGCCGTGGTT-3'
Adcy 1	5'-ATATCCGAGAGAATCAAGCCT-3'	5'-GTCCACATCACAAAGACGACC-3'
Adcy 2	5'-TCGATCTCCTCCCGCTCT-3'	5'-CATCATTCTGCTCCACACCCAT-3'
Adcy 3	5'-TACCACTTTGCGGCTCACTC-3'	5'-GACATCATCCTTTTCGTGCTCT-3'
Adcy 6	5'-GTGCCCCGTGTTCTTCGTCT-3'	5'-CCAGGCCAAAATCAAATGCAG-3'
Adcy 8	5'-CATTTCCTCAGGCCCAACAC-3'	5'-AGATCCAGAACGAAGCACGA-3'
Cyclophilin A	5'-CGTGGCCAACGATAAGAAGAA-3'	5'-GTCTCCACCCTGGATCATGAA-3'

Supplementary Table 3: RT-PCR and PCR primers

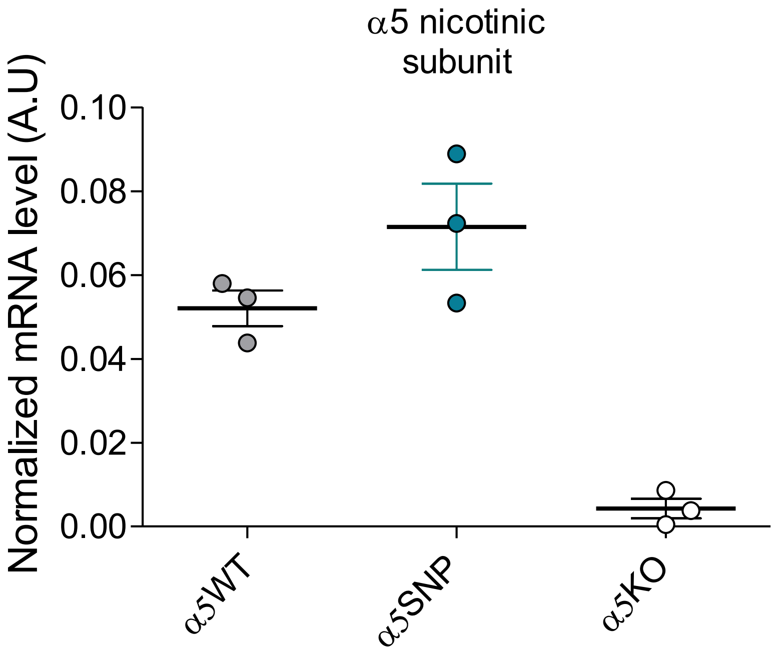

Supplementary Figure 1: Gene expression levels of α5 nAChR subunit in isolated epithelial basal cells from WT, α5SNP and KO mice.

Total RNA was extracted from csBEC (6 mice) using a RNeasy mini kit (Qiagen, 74104). cDNA was synthetized with M-MLV reverse transcriptase (Invitrogen, 28025-013) and random hexamer primers (ThermoFisher, SO142). qPCR was carried out in a LightCycler 480 instrument (Roche Applied Science) using the KAPA SYBR FAST qPCR Master Mix (KapaBiosystems, KK4611) and specific primers detailed in **Table S3**. All reactions were run in duplicate, repeated 3 times and results were normalized to cyclophilin A expression. Data are expressed as mean ± SEM.

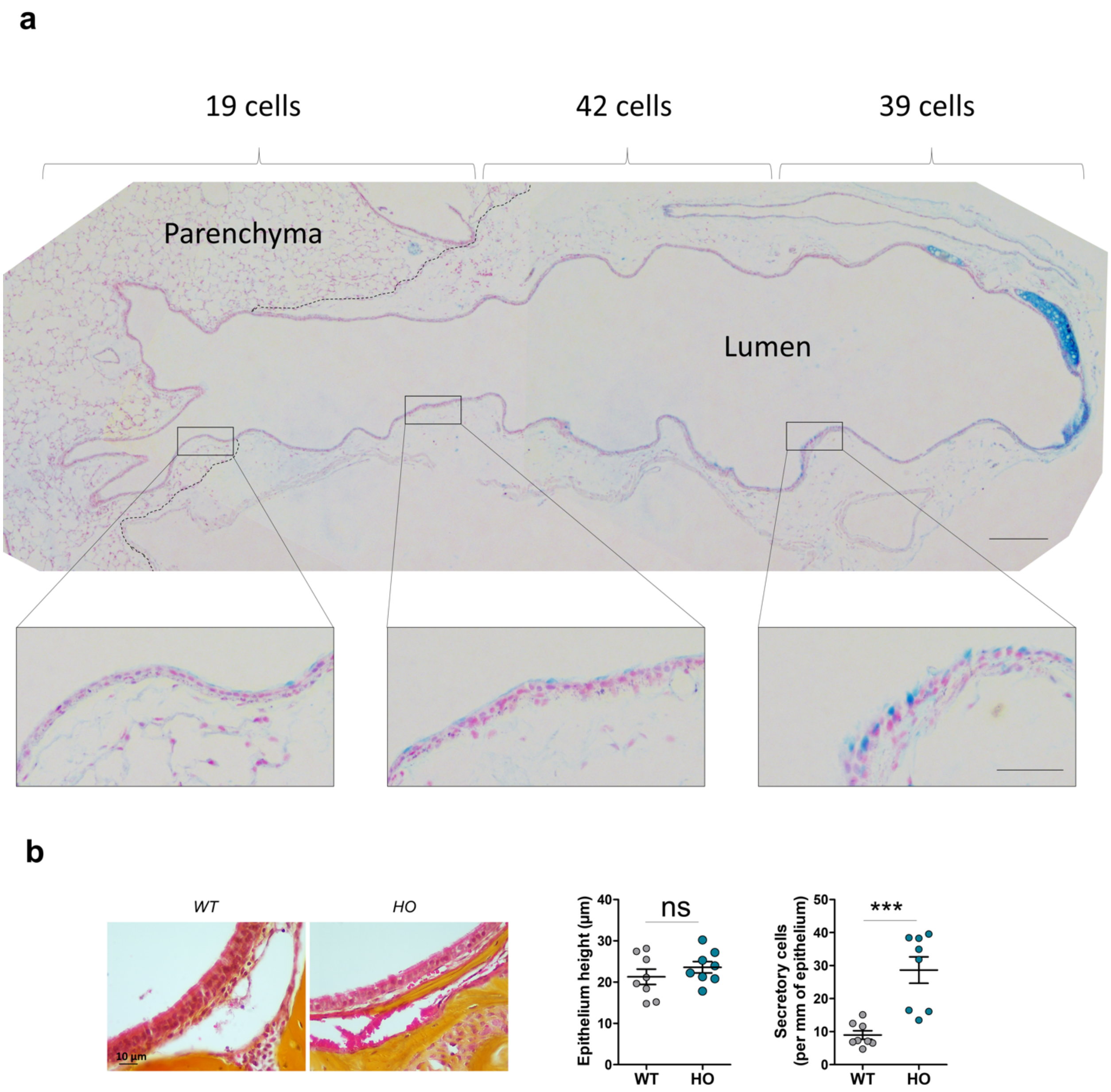

Supplementary Figure 2: Distribution of goblet cells in respiratory epithelia of α5SNP HO mice.

a Example of an Alcian blue staining of an extra-lobular bronchus in a 24 wo α5SNP HO mouse. The dotted line represents the limit of the lung. Quantification of Alcian blue-positive cells in the three areas shows a rapid decline of mucus cell numbers within the lung. Scale bars: main picture: 200 µm; magnification: 50 µm. **b** Quantification of epithelium height and goblet cells in nasal respiratory epithelia of WT and HO α5SNP 54 wo mice (n=8 mice per group). Data are expressed as mean ± SEM. For statistical analyses, values are compared to WT mice. *** $p<0.001$, Mann-Whitney two-sided test.

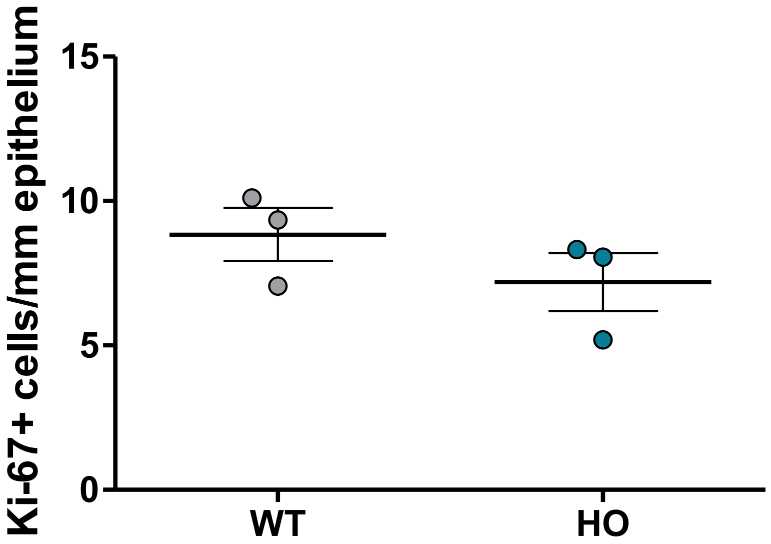

Supplementary Figure 3: α5SNP does not alter cell proliferation in broncho-alveolar junctions.

Quantification of Ki-67+ cells in broncho-alveolar junctions for α5SNP HO (n=3) and WT mice (n=3). Box plots show the medians and whiskers correspond to maximal and minimal values. For statistical analyses, values are compared to the appropriate control (WT). Data are expressed as mean ± SEM. For statistical analyses, values are compared to WT mice.

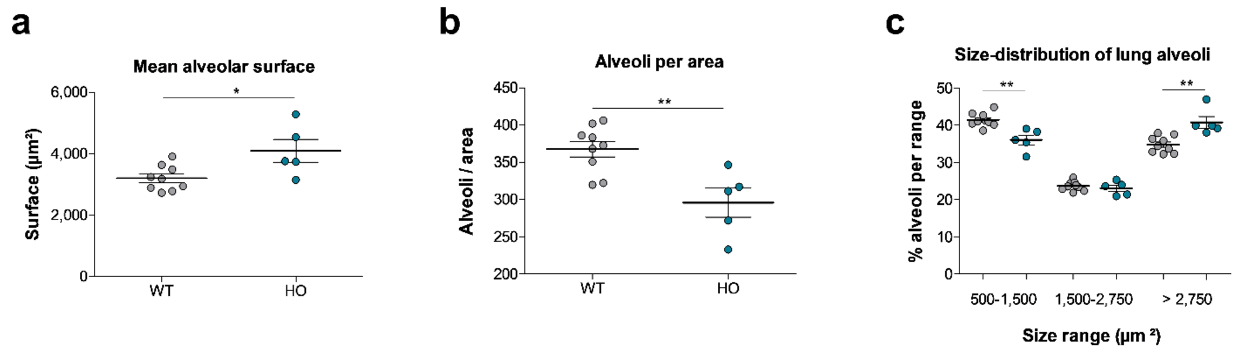

Supplementary Figure 4: More severe emphysematous lung lesion in α5SNP HO mice confirmed by image analysis.

Spontaneous emphysema in 54 wo HO α5SNP mice was confirmed by measuring the mean alveolar surface **a**, the mean number of alveoli per area **b**, and the percentage of alveoli in each class of alveolus surface of 500-1500 μm^2, 1500-2750 μm^2 and more than 2750 μm^2 **c** (n=9 WT and 5 HO). In **a-c**, dot plots show the means ± SEM. * $p < 0.05$; ** $p < 0.01$, Mann-Whitney two-sided test.

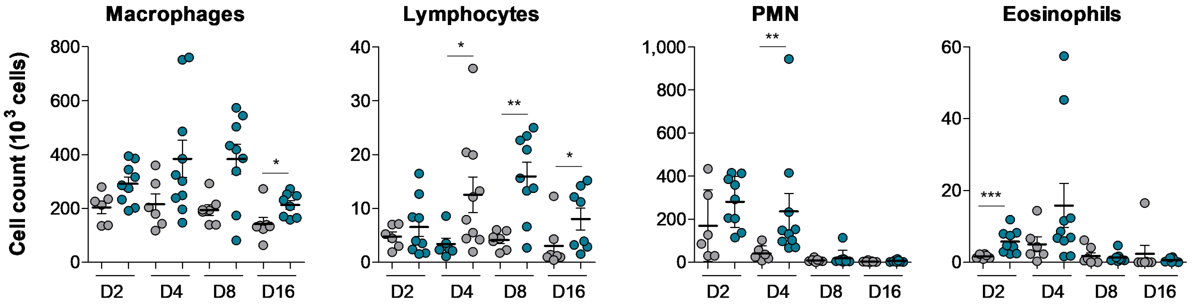

Supplementary Figure 5: Inflammatory cell recruitment is increased in α5SNP HO mice after oxidative stress.

Evolution of macrophages, lymphocytes, polymorphonuclear (PMN) cells and eosinophils in BALF from HO α5SNP and WT 12 wo females, at 2 days (D2), D4, D8 and D16 after instillation of a polidocanol solution (n=6 WT, grey circles and 9 HO, blue circles per time points). Data are expressed as mean ± SEM. For statistical analyses, values are compared to the WT mice for the same day. * $p < 0.05$; ** $p < 0.01$; *** $p < 0.001$ (Mann Whitney two-sided test).

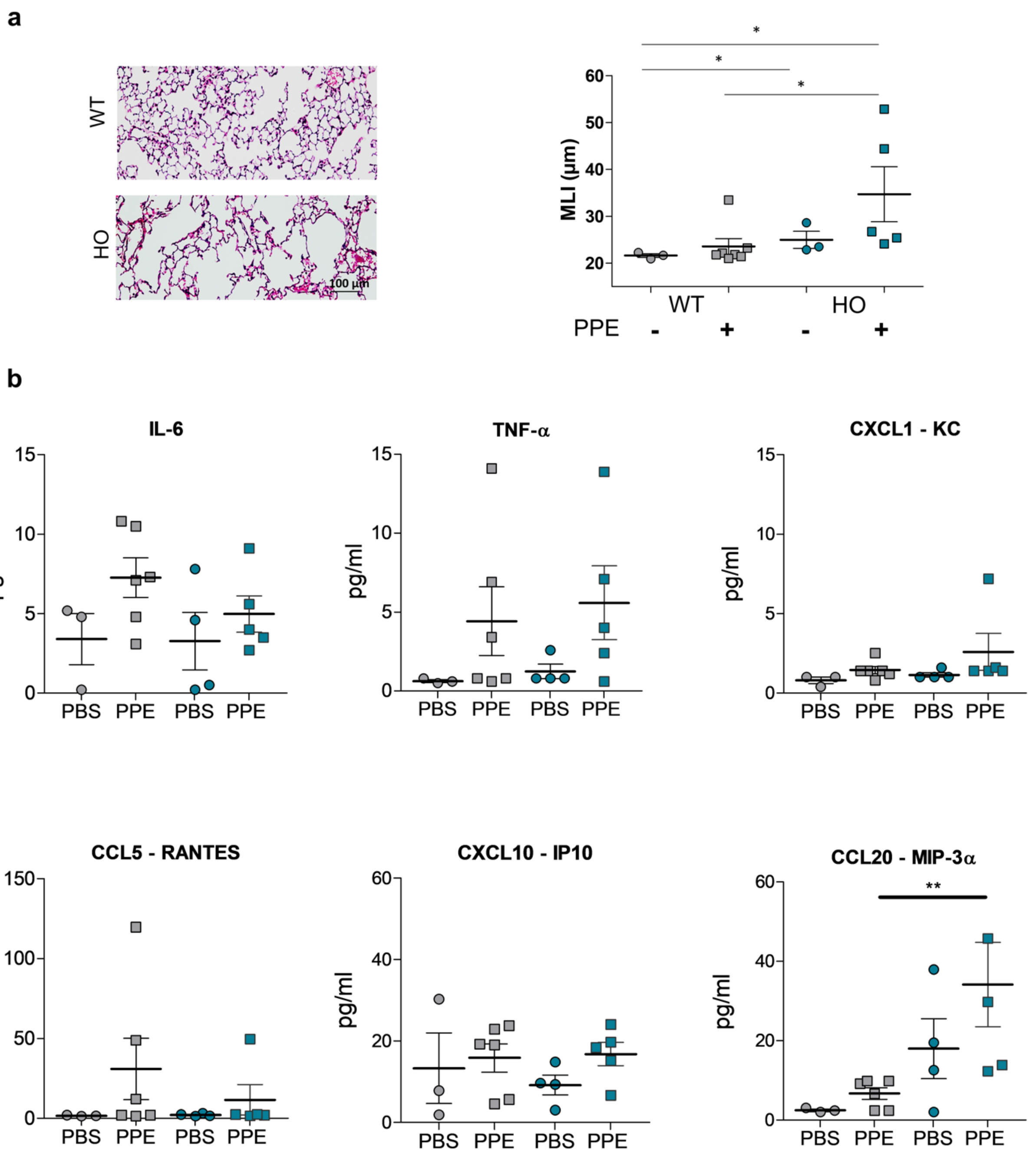

Supplementary Figure 6: Evaluation of porcine pancreatic elastase (PPE)-induced emphysema and inflammation in WT and α5SNP mice.

a Quantification of MLI after instillation of either PBS or PPE (n=10 WT and 8 HO). **b** Expression of cytokines IL-6, TNF-α, CXCL1, CCL5, CXCL10 and CCL20 in BALF from WT (grey, n=9) and α5SNP HO (blue, n=9) mice treated with PPE (squares) or PBS (circles). Data are expressed

as mean ± SEM. For statistical analyses, values are compared to WT mice. *$p<0.05$; ** $p < 0.01$ (Mann-Whitney two-sided test).

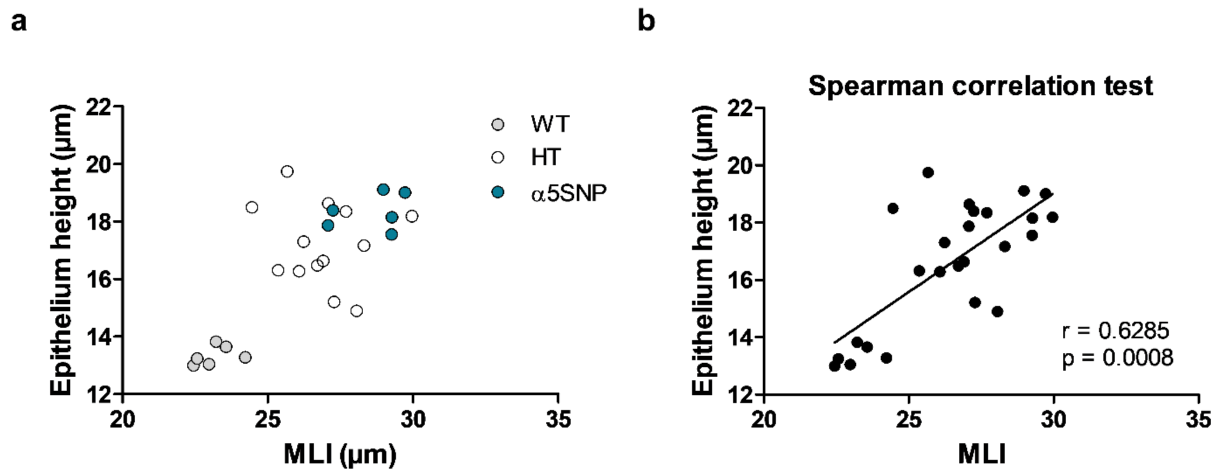

Supplementary Figure 7: Correlation between emphysema and bronchiolo-alveolar epithelium height after an oxidative stress in WT and HT and HO αSNP mice.

a Emphysematous lesions in cumene exposed mice were assessed by MLI measurement, and epithelium height was also determined at bronchiolo-alveolar junctions as shown in **Fig. 3a** and **b**. **b** Spearman correlation two-sided test showing a positive correlation between emphysema and epithelial height.

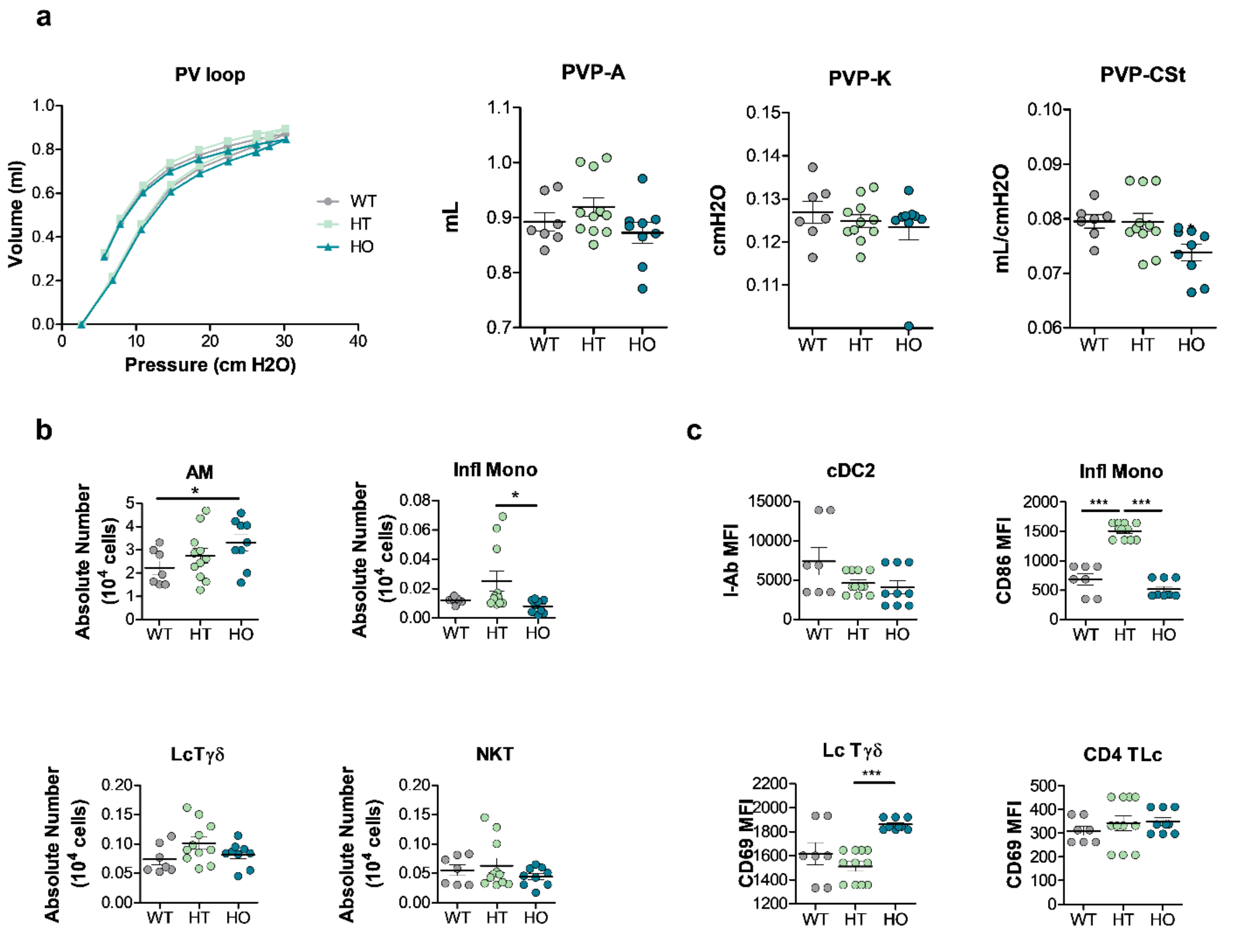

Supplementary Figure 8: Basal respiratory function and inflammatory cells in BALF of WT and HT and HO α5SNP mice.

a Basal lung function in WT (n=7) grey circles, HT (n=11) green circles and HO (n=9) blue circles, α5SNP mice nasally instilled with PBS 7, 5 and 3 days before analysis. Parameters A and K and Cst expressed as mean ± SEM. The number of alveolar macrophages (CD11c+, F4-80+, Siglec F+), inflammatory monocytes (CD11c-, F4-80+, Ly-6C+, CCR2+, CD64+), Tγδ lymphocytes (CD5+, TCRγδ+) and NKT cells (CD5+, TCRβ+, CD1d tetramer+) was measured in BALF by flow cytometry in **b**, and activation of cDc2 cells (I-Ab MFI), inflammatory monocytes (CD86 MFI), Tγδ lymphocytes and CD4+ T cells (CD69 MFI) was determined in **c**. In a-c, dot plots show the means ± SEM. * p< 0.05; ** p< 0.01 (Mann-Whitney two-sided test).

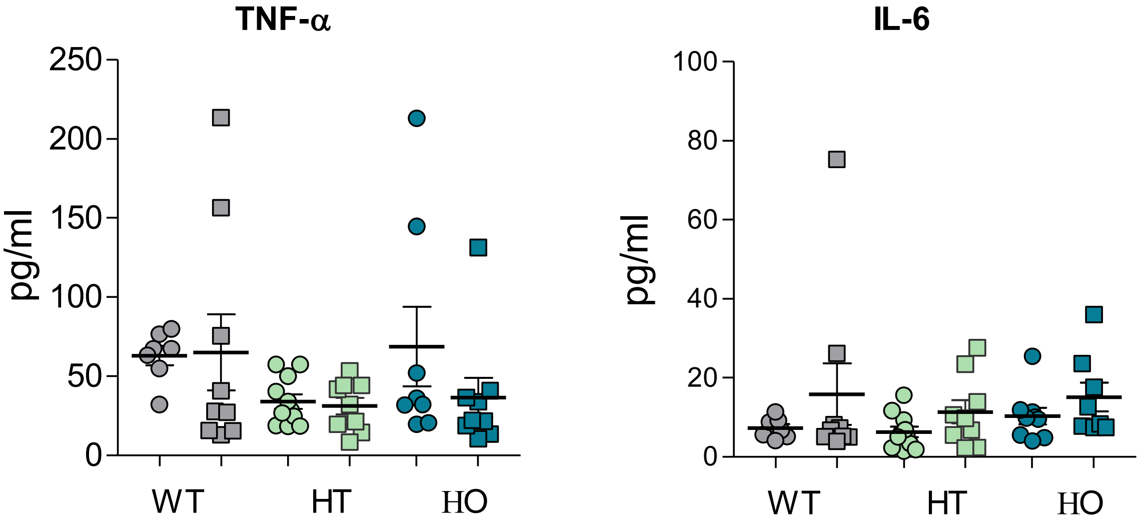

Supplementary Figure 9: Cytokine concentrations after an oxidative stress in WT, HT and HO αSNP mice.

IL-6 and TNF-α concentrations in the BAL from WT (n=16), heterozygous (HT) (n=20) and homozygous (HO) (n=18) α5SNP mice exposed to cumene hydroperoxide-induced oxidative stress (squares) or to PBS (circles). Data are expressed as mean ± SEM.

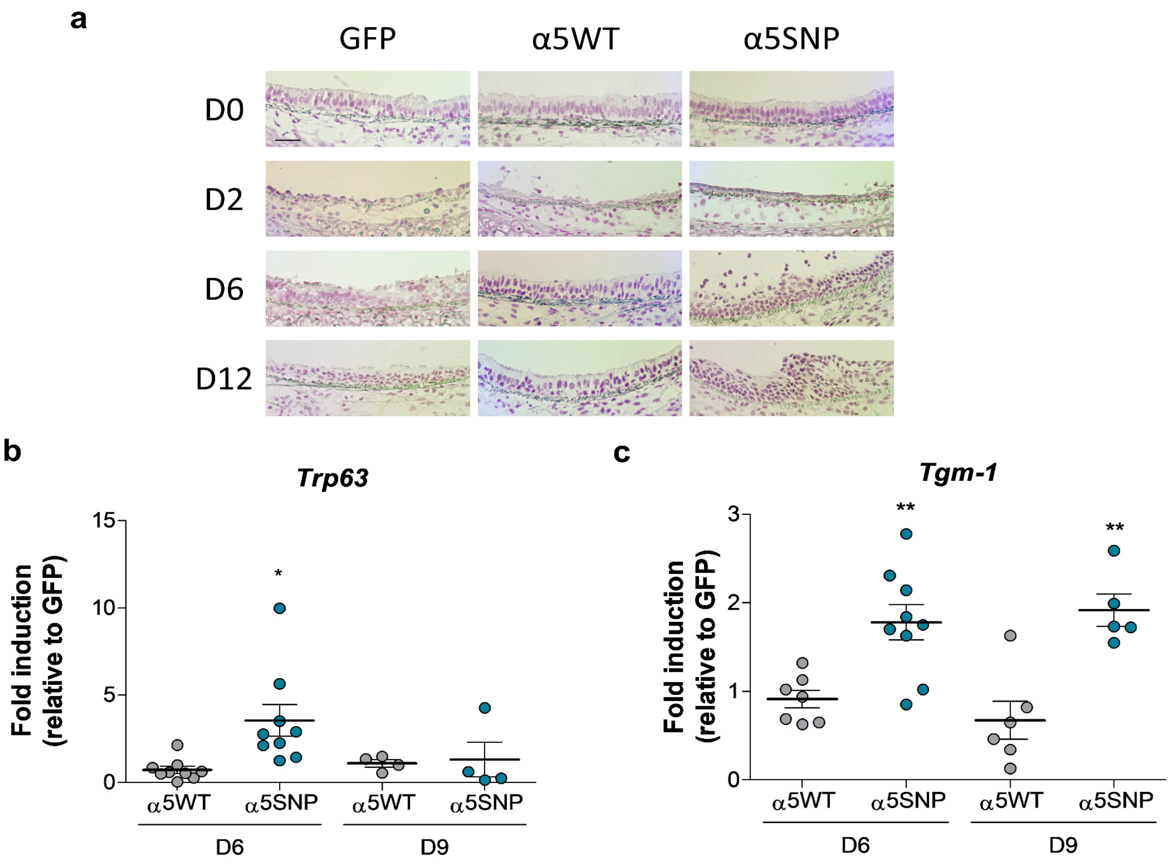

Supplementary Figure 10: Effect of *Chrna5* genotype on epithelium morphology during repair, and on squamous metaplasia marker expression.

a Kinetics of epithelial regeneration after a polidocanol lesion in GFP, α5WT or α5SNP LV mice. Micrographs representative from three experiments (Scale bar=20 μm). **b** Expression of squamous metaplasia marker *Trp63* in tracheas on D6 (n=9 mice in each group) and D9 (n=4 mice in each group) of epithelial repair. **c** Expression of squamous metaplasia marker *Tgm-1* in tracheas on D6 (n=7 α5WT and n=9 α5SNP LV mice) and D9 (n=6 α5WT and n=5 α5SNP LV mice) of epithelial repair. Results are presented as mean ± SEM of tracheal mRNA expression related to GFP control mice and values for α5SNP mice were compared to α5WT with *$p < 0.05$; **$p < 0.01$, Mann-Whitney two-sided test.

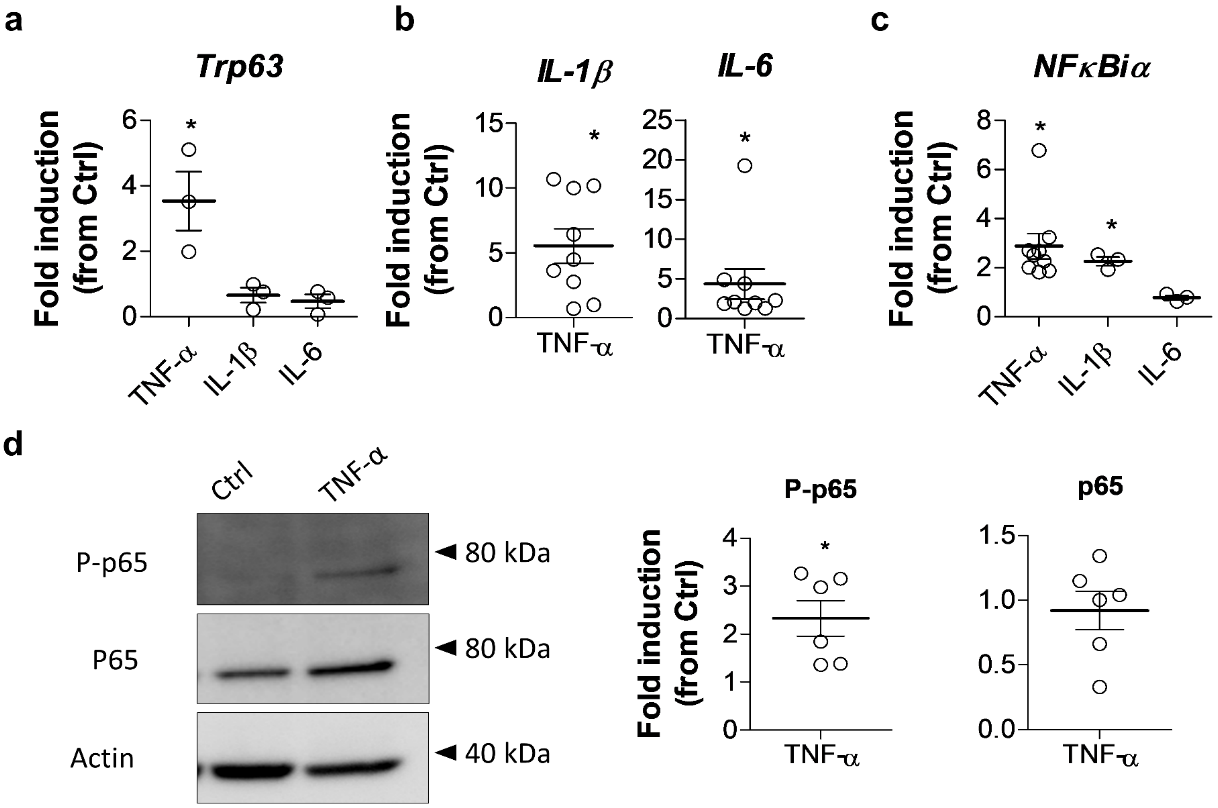

Supplementary Figure 11: Effect of *Chrna5* genotype on epithelium morphology during repair, and on squamous metaplasia marker expression.

a Kinetics of epithelial regeneration after a polidocanol lesion in GFP, α5WT or α5SNP LV mice. Micrographs representative from three experiments (Scale bar=20 µm). **b** Expression of squamous metaplasia marker *Trp63* in tracheas on D6 (n=9 mice in each group) and D9 (n=4 mice in each group) of epithelial repair. **c** Expression of squamous metaplasia marker *Tgm-1* in tracheas on D6 (n=7 α5WT and n=9 α5SNP LV mice) and D9 (n=6 α5WT and n=5 α5SNP LV mice) of epithelial repair. Results are presented as mean ± SEM of tracheal mRNA expression related to GFP control mice and values for α5SNP mice were compared to α5WT with *p < 0.05; **p < 0.01, Mann-Whitney two-sided test.

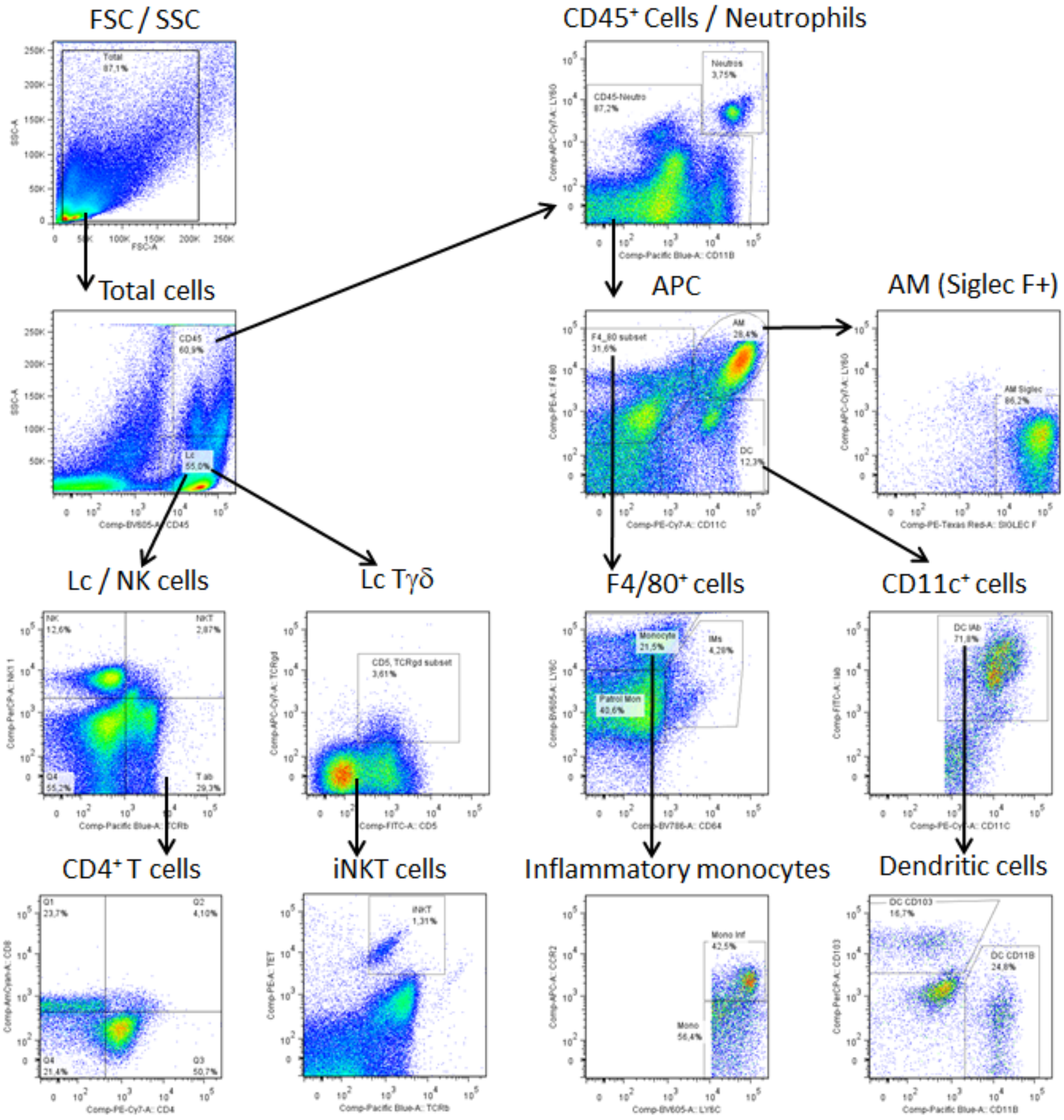

Supplementary Figure 12: gating strategy for neutrophils, antigen-presenting cells (APC) and the major populations of lymphocytes (Lc) in the lung.

The percentages of natural killer (NK), invariant NKT cells (iNKT) and T Lc with a TCRgd was determined. Among APC, we identified alveolar macrophages (AM), inflammatory monocytes,

patrolling monocytes, interstitial macrophages (IM) and conventionnal dendritic cells (cDC)1 and 2 as CD103+ and CD11b+ cells, respectively.

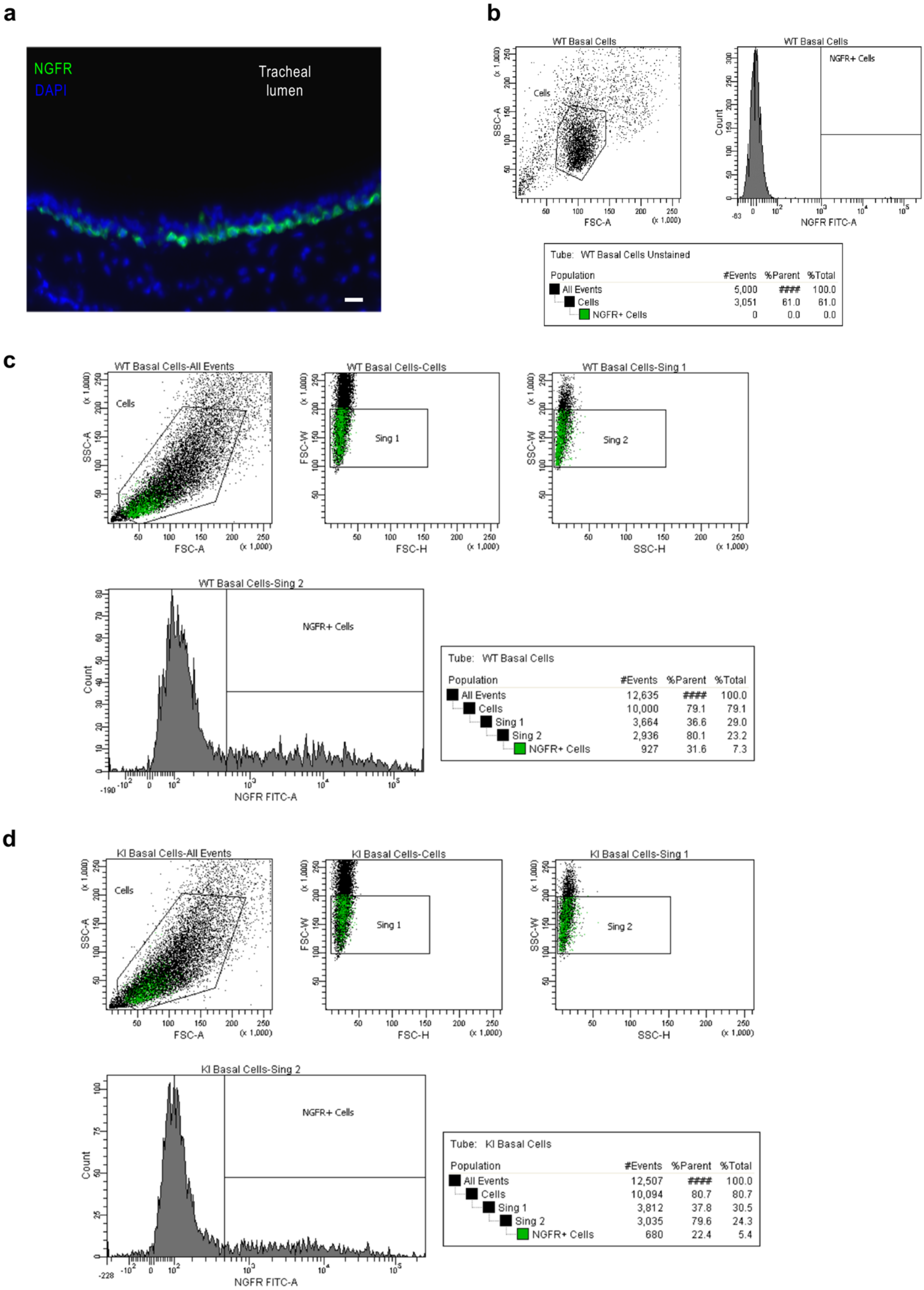
a
NGFR
DAPI
Tracheal lumen
b
WT Basal Cells
Cells
SSC-A
FSC-A
NGFR+ Cells
Count
NGFR FITC-A
Tube: WT Basal Cells Unstained
Population #Events %Parent %Total
All Events 5,000 #### 100.0
Cells 3,051 61.0 61.0
NGFR+ Cells 0 0.0 0.0
c
WT Basal Cells-All Events
WT Basal Cells-Cells
WT Basal Cells-Sing 1
Sing 1
Sing 2
FSC-H
SSC-H
FSC-W
SSC-W
WT Basal Cells-Sing 2
Tube: WT Basal Cells
Population #Events %Parent %Total
All Events 12,635 #### 100.0
Cells 10,000 79.1 79.1
Sing 1 3,664 36.6 29.0
Sing 2 2,936 80.1 23.2
NGFR+ Cells 927 31.6 7.3
d
KI Basal Cells-All Events
KI Basal Cells-Cells
KI Basal Cells-Sing 1
KI Basal Cells-Sing 2
Tube: KI Basal Cells
Population #Events %Parent %Total
All Events 12,507 #### 100.0
Cells 10,094 80.7 80.7
Sing 1 3,812 37.8 30.5
Sing 2 3,035 79.6 24.3
NGFR+ Cells 680 22.4 5.4

Supplementary Figure 13: Flow cytometric analyses (FACS) and sorting of mouse tracheal basal epithelial cells.

Flow cytometric analyses (FACS) and sorting of mouse tracheal basal epithelial cells. This figure presents immunohistostaining (**a** Scale bar = 20 μm) and FACS **b** data that support NGFR as a surface marker of airway basal cells in WT **c** and α5SNP HO **d** mice.

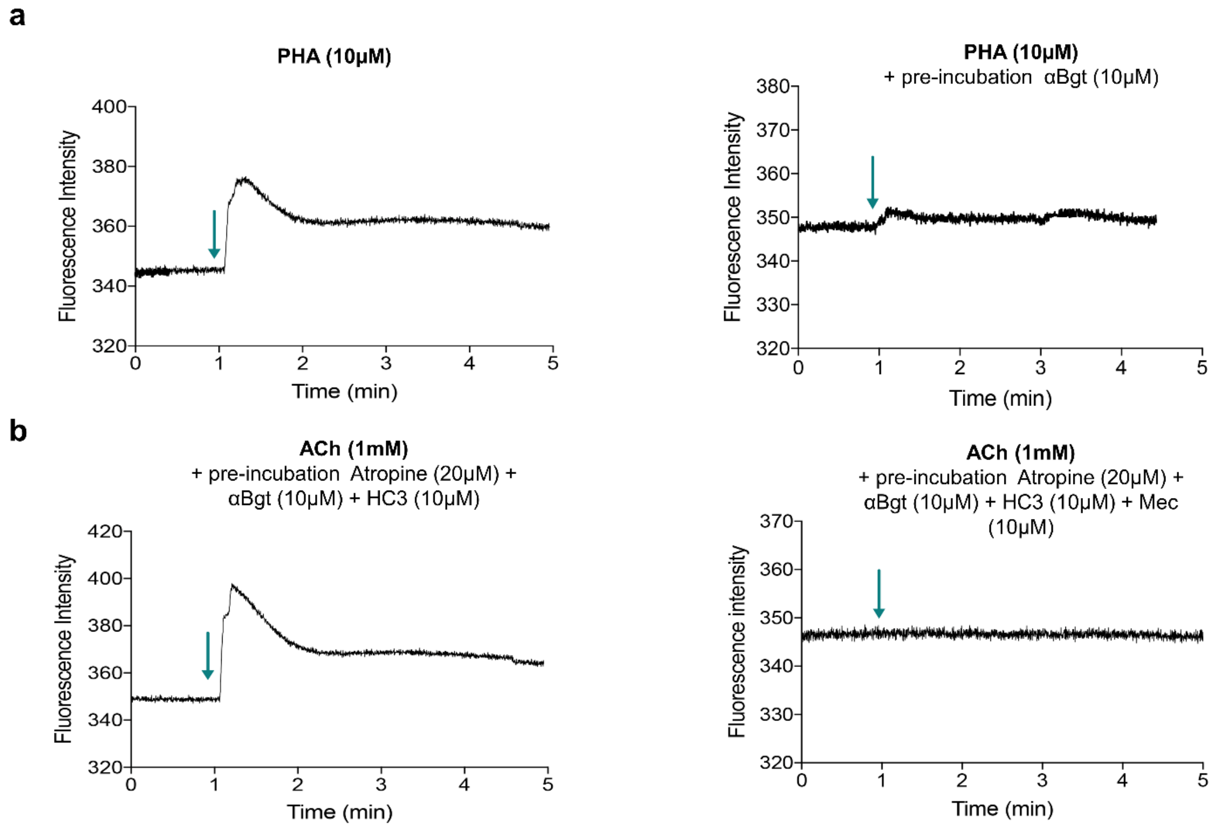

Supplementary Figure 14: Control conditions for calcium imaging on csBEC from LV mice. Airway basal cells express α7 nAChRs and muscarinic receptors (mAChRs). **a** Validation of blocking condition of α7 nAChR: Measurement of fluorescence intensity representing Ca^{2+} entry in response to an application of 10µM PHA 568487[2] (α7 nAChR selective agonist), green arrow, in basal cells without pre-incubation (left panel). Response blocked by 10µM α-Bungarotoxin (αBgt, α7 nAChR antagonist) pre-incubation of basal cells (right panel). **b** Validation of condition to measure fluorescence intensity representing Ca^{2+} entry only due to heteropentameric nAChR, in response to an application of 1mM ACh, green arrow. In csBCE pre-incubated in presence of 10µM αBgt, 20µM atropine and 10µM hemicholinium3 (HC3) to block α7 nAChR, mAChRs and

the uptake of choline (right panel). Response blocked by addition of 10μM mecamylamine, a non-competitive nAChR antagonist[3], during pre-incubation (right panel).

Supplemental references

1. Lesage, J. *et al.* Zonula occludens-1 / NF- k B / CXCL8 : a new regulatory axis for tumor angiogenesis. *FASEB J.* **31**, 1678–1688 (2018).
2. Walker, D. P. *et al.* Design, synthesis, structure-activity relationship, and in vivo activity of azabicyclic aryl amides as α7 nicotinic acetylcholine receptor agonists. *Bioorganic Med. Chem.* **14**, 8219–8248 (2006).
3. Bacher, I., Wu, B., Shytle, D. R. & George, T. P. Mecamylamine – a nicotinic acetylcholine receptor antagonist with potential for the treatment of neuropsychiatric disorders. *Expert Opin. Pharmacother.* **10**, 2709–2721 (2009).
