# Supplementary material for: An innate contribution of human nicotinic receptor polymorphisms to COPD-like lesions
Source: Nat Commun. 2021 Nov 4;12:6384. doi: 10.1038/s41467-021-26637-6 (PMC8568944; doi:10.1038/s41467-021-26637-6)
Supplement: Supplementary file 3 — Reporting Summary [file 41467_2021_26637_MOESM3_ESM.pdf]

nature research

Corresponding author(s): Dormoy, Maskos

Last updated by author(s): 31/8/21

Reporting Summary

Nature Research wishes to improve the reproducibility of the work that we publish. This form provides structure for consistency and transparency in reporting. For further information on Nature Research policies, see our Editorial Policies and the Editorial Policy Checklist.

Statistics

For all statistical analyses, confirm that the following items are present in the figure legend, table legend, main text, or Methods section.

n/a	Confirmed	
☐	☒	The exact sample size (n) for each experimental group/condition, given as a discrete number and unit of measurement
☐	☒	A statement on whether measurements were taken from distinct samples or whether the same sample was measured repeatedly
☐	☒	The statistical test(s) used AND whether they are one- or two-sided<br>*Only common tests should be described solely by name; describe more complex techniques in the Methods section.*
☐	☒	A description of all covariates tested
☒	☐	A description of any assumptions or corrections, such as tests of normality and adjustment for multiple comparisons
☐	☒	A full description of the statistical parameters including central tendency (e.g. means) or other basic estimates (e.g. regression coefficient) AND variation (e.g. standard deviation) or associated estimates of uncertainty (e.g. confidence intervals)
☐	☒	For null hypothesis testing, the test statistic (e.g. F, t, r) with confidence intervals, effect sizes, degrees of freedom and P value noted<br>*Give P values as exact values whenever suitable.*
☒	☐	For Bayesian analysis, information on the choice of priors and Markov chain Monte Carlo settings
☒	☐	For hierarchical and complex designs, identification of the appropriate level for tests and full reporting of outcomes
☐	☒	Estimates of effect sizes (e.g. Cohen's d, Pearson's r), indicating how they were calculated

Our web collection on statistics for biologists contains articles on many of the points above.

Software and code

Policy information about availability of computer code

Data collection	- Emphysema and epithelium height were quantified using custom codes in ImageJ v1.51J software (Methods/Emphysema quantification and Methods/ Epithelium heigt measure) which are available upon request. Other softewares used during this study are detailed above.<br>- segmented line selection tool: ImageJ v1.51J software<br>- Proliferation assays : Andor iQ2 and Opera QEHS softwares.<br>- qPCR data collection : LightCycler 480<br>- Genotyping gel analysis : Aïda software (Raytest).<br>- Microscopy : NIS-Elements (Nikon) or Zen Version 8.1 (Zeiss)<br>- Measurement of lung function : FlexiWare v7.7 (Scireq Inc.)<br>- Flow cytometry : FlowJo v7.6.5 or FACSDiva (see methods /Flow cytometry or /Cytokine quantification or /FACS sorting procedure)
Data analysis	- Image treatments : Fiji v1.52d software.<br>- FACS quantifications : FACSDiva software (BD Bioscience)<br>- CBA analysis : FCAP software v3.0.1 (BD Bioscience)<br>- Quantification of qPCR data : LightCycler 480 software (release 1.5.0)<br>- Western Blot analysis : MultiGauge v3.0 (Fujifilm).<br>- Statistical analysis of epithelial phenotype in human samples : Stata software (version 10.0, Stata corporation).<br>- Graph creation and statistical analysis : GraphPad Prism 7.0a software (Paris) and v_5 (Reims)

For manuscripts utilizing custom algorithms or software that are central to the research but not yet described in published literature, software must be made available to editors and reviewers. We strongly encourage code deposition in a community repository (e.g. GitHub). See the Nature Research guidelines for submitting code & software for further information.

Data

Policy information about availability of data

All manuscripts must include a data availability statement. This statement should provide the following information, where applicable:

- Accession codes, unique identifiers, or web links for publicly available datasets
- A list of figures that have associated raw data
- A description of any restrictions on data availability

The authors declare that all data supporting the findings of this study are available within the article and its Supplementary Information files. Source data are provided with this paper. Any remaining raw data will be available from the corresponding authors upon reasonable request.

Field-specific reporting

Please select the one below that is the best fit for your research. If you are not sure, read the appropriate sections before making your selection.

☒ Life sciences ☐ Behavioural & social sciences ☐ Ecological, evolutionary & environmental sciences

For a reference copy of the document with all sections, see nature.com/documents/nr-reporting-summary-flat.pdf

Life sciences study design

All studies must disclose on these points even when the disclosure is negative.

Sample size	- Polyp study: No sample-size calculation was performed. According to French Ethics Committee authorization 2016-A00242-49, we included in our study polyp samples from all patients undergoing polypectomy between 2011-2016, leading to 213 samples.<br>- Mice study: The number of animals used to measure intracellular calcium, proliferation test or scratch wound healing assay were based on the expected number of basal cells which were isolated from individual animals and have been published by this laboratory (Tournier J-M. et al., Am. J. Pathol. 2006, Maouche K. et al., Am. J. Pathol., 2009). Typically cell yield/mouse from trachea is 2 x10.5 cells. Number of mice / group/ independent experiment was approved by the Animal Ethics Committee and is available in figure legends.
Data exclusions	- Polyp study: of the 213 polyps analysed in our study, 64 presented an epithelial abrasion higher than 70% and were eliminated from the analysis. 24 patients were smokers and were excluded from the analysis. Abrasion was an exclusion criteria pre-established, while smokers have been excluded because the effective was too small to power the statistics of association regarding this criteria. - Mice study: for BAL analysis, mice were excluded if blood was found in BAL samples. For mucinous cells quantification, mice were excluded if the extralobar part of main bronchus was not disposable.
Replication	- Polyp study: histological quantifications were performed by two histo-pathologists independently and the results were compared. If the quantitative assessment differed more than 5%, the images were analysed an additional time by the two pathologists together to confirm the values. - In vitro studies: all attempts of replication were successful. The minimum replication was duplicate and the numbers are mentioned in the manuscript.<br>- mice studies: for all experiments involving measurements in mice and their biological specimen, and in accordance with the 3R, the appropriate number of animal was analyzed (n>3) to warrant the reproducibility of the experiment findings. All attempts at replication were successful.
Randomization	- Polyp study: not applicable. All biological specimen for each group defined by the genotypes were included unless the exclusion criteria were met. - Mice study: mice from all our experiments were age- and sex-matched.
Blinding	- Polyp study: histological quantifications were blindly performed by two histo-pathologists. - Mice study: investigators were blinded to mice group allocation during data collection and analyses of samples.<br>- In vitro studies: investigators were blinded to in vitro group allocation during data collection and analyses of samples.

Reporting for specific materials, systems and methods

We require information from authors about some types of materials, experimental systems and methods used in many studies. Here, indicate whether each material, system or method listed is relevant to your study. If you are not sure if a list item applies to your research, read the appropriate section before selecting a response.

Materials & experimental systems

n/a	Involved in the study
	☒ Antibodies
☒	☐ Eukaryotic cell lines
☒	☐ Palaeontology and archaeology
	☒ Animals and other organisms
	☒ Human research participants
☒	☐ Clinical data
☒	☐ Dual use research of concern

Methods

n/a	Involved in the study
☒	☐ ChIP-seq
	☒ Flow cytometry
☒	☐ MRI-based neuroimaging

Antibodies

Antibodies used

Methods/ Supplementary table 2. Flow cytometry:
FITC-I-Ab (Miltenyi Biotech, 130-102-168, M5/114.15.2, 5161107010, 1/200), PE-F4/80 (Miltenyi Biotech, 130-102-422, REA126, 5151203251, 1/200), PerCP Cy5.5 CD103 (BD Biosciences, 563637, M290, 7104716, 1/300), PE-Cy7-CD11c (BD Biosciences, 558079, HL3, 8083701, 1/500), APC-CCR2 (R&D systems, FAB5538A, 475301, ABLE0314121, 1/200), AF700-CD86 (BD Biosciences, 560581, GL1, 7034805, 1/100), APC-H7-Ly6G, BD Biosciences, 560600, 1A8, 6263521, 1/500), V450-CD11b (BD Biosciences, 560455, M1/70, 5322624, 1/300), V500-CD45 (Miltenyi Biotech, 130-102-412, 30F11, 5150423227, 1/300), BV605-Ly6C (Biolegend, 128036, HK1.4, B245767, 1/300), BV786-CD64 (BD Biosciences, 741024, X54-5/7.1, 7319699 , 1/500), PE-CF594-SiglecF (BD Biosciences, 562757, E50-2440, 7128860, 1/300), FITC-CD5 (Miltenyi Biotech, 130-102-574, 53.7.3, 5141118262, 1/200), Tetramer mCD1d 167ms (NIH facility, 30663, 2016-06-26, 1/500), PerCP Cy5.5 NK1.1 (Miltenyi Biotech, 130-103-963, PK136, 5141202377, 1/300), PE-Cy7 CD4 (Miltenyi Biotech, 130-102-411, GK1.5, 5150601111, 1/500), APC-CD25 (Miltenyi Biotech, 130-102-550, PC61, 5141202369, 1/200), AF700-CD69 (BD Biosciences, 561238, H12F3, 7054759, 1/500), APC-Vio770 TCRγδ (Miltenyi Biotech, 130-104-016, GL3, 5171122319, 1/100), V450-TCRb (Miltenyi Biotech, 130-104-815, REA318, 5171122321, 1/100), V500-CD8 (BD Biosciences, 130-109-252, REA601, 5161107165, 1/500), BV605CD45 (Biolegend, 103140, 30F11, B235438, 1/300), FITC IgG1 (BD Biosciences, 555749, MOPC21, 5225509, 1/100), IgG- isotype control (Abcam, ab37415, 18μg/mL), NGFR (Abcam, ab8875, 18 μg/ml), GFP (Invitrogen, A-6455, 1/750), Goat anti-rabbit AF488 (Abcam, ab150077, 1/1000), Goat anti-rabbit AF633 (Thermo Fisher Sc, A-21070, 1/1000).
Immunohistochemistry:
CC10 (Abcam, ab40873, GR21852011, 1/3200), Acetylated tubulin (Sigma-Aldrich, T6793, 6-11B-1, 034M4828, 1/1000), GFP (Thermo Fisher Sc, A-6455, 1826342, 1/1000), Adenylate cyclase-1 (Santa Cruz, Sc-25743, L1208, 1 μg/ml), Adenylate cyclase-3 (Santa Cruz, Sc-32114, B1413, 1 μg/ml), Adenylate cyclase-8 (Santa Cruz, Sc-20764, C1811, 1 μg/ml), CK5 (Abcam, ab53121, 5μg/ml), Pan-cytokeratin (Elabscience, E-AB-33599, DK9774, 10 μg/ml), CD45 (Dako, M0701, 2B11+PD7/26, 1/50), CD68-KP1 (Dako, M0814, KP1, 1/400), NGFR (Abcam, ab8875, 10 μg/ml), IgG-isotype control (Abcam, ab37415, 10μg/mL), Goat anti-rabbit AF488 (Molecular Probes, A11008, 1885240, 2 μg/ml), Donkey anti-rabbit AF488 (Molecular Probes, A21206, 1796375, 2 μg/ml), Goat anti-rabbit AF594 (Molecular Probes, A11012, 1084427, 2 μg/ml), Donkey anti-goat AF594 (Molecular Probes, A11058, 1736986, 2 μg/ml).
Immunoblotting:
Phospho-p65 (Cell Signaling #3031, 1/1000), p65 (Santa-Cruz, Sc-372, 1/1000), Actin (Sigma-Aldrich, A2066, 1/1000).

Validation

For all antibodies, validation is provided at the manufacturer's website and concentrations and conditions were adapted to our general protocols. The validation statements include (i) for flow cytometry: unstained, positive, negative, isotype, viability, Fc-blocking, fluorescence minus one (FMO), single-staining controls, and KO cell lines whenever these are available; (ii) for immunohistochemistry: staining multi-normal human tissue microarrays (TMAs), multi-tumor human TMAs, and rat or mouse TMAs during antibody development; (iii) for immunoblotting: lysates from cells or tissues that were identified to express the protein of interest are used, several controls in the same western blot experiment, including positive lysate and negative lysate are performed, when possible knock-out (KO) cell lines as true negative control are used. In addition, for FACS analysis and immunohistochemistry, cells were incubated with IgG-isotype control (ab37415) to confirm the absence of signal.

Animals and other organisms

Policy information about studies involving animals; ARRIVE guidelines recommended for reporting animal research

Laboratory animals

Methods/Mice. Animals are maintained in different animal facilities on the Institut Pasteur campus, each of which is licensed by the French Ministry of Agriculture. Each facility has central air conditioning equipment which maintains a constant temperature of 22 ± 2° C (rodents). In the BIME-EOPS facility, the air is H12 filtered and renewed at least 20 times per hour in animal rooms. Hygrometry is not regulated. Animals are housed in individually ventilated cages (IVCs) which comply with European regulations in terms of floor surface per animal.Mice were group housed in the central animal facility. Light/dark cylcle is 12h each, lights on at 7am. Mice are on a 100% C57BL/6J genetic background. Male and femaile mice used in the study were aged 6 to 54 weeks, as indicated in the manuscript.

Wild animals

The study did not involve wild animals.

Field-collected samples

The study did not involve field-collected samples.

Ethics oversight

Ethics Committee of the University of Reims Champagne-Ardenne (approvals 56-2012-3 and 56-2012-4), the Institut Pasteur CETEA (01828.02), and by the French Ministry of Research (approvals 4362-2016030315538085, 2016120715475887 and 2017100512017052415271111v1)

Note that full information on the approval of the study protocol must also be provided in the manuscript.

Human research participants

Policy information about studies involving human research participants

Population characteristics	Methods / Human nasal polyps and Supplementary Table 1. The covariate-relevant population characteristics of the human research participants collected were the following: age, gender, CHRNA5 genotype, smoking status, and presence/absence of allergy/asthma.
Recruitment	Methods / Human nasal polyps. Human nasal polyps were obtained from 213 patients undergoing nasal polypectomy/sinus surgery once the approval of the French Ehtics Committee was received (Comité de Protection des Personnes Est 1 – authorization 2016-A00242-49) according to the French law. All the polyps that were collected and that could be genotyped have been used so that no biases could be implemented during recruitment.
Ethics oversight	French Ethics Committee (Comité de Protection des Personnes Est 1 – authorization 2016-A00242-49)

Note that full information on the approval of the study protocol must also be provided in the manuscript.

Flow Cytometry

Plots

Confirm that:

- [x] The axis labels state the marker and fluorochrome used (e.g. CD4-FITC).
- [x] The axis scales are clearly visible. Include numbers along axes only for bottom left plot of group (a 'group' is an analysis of identical markers).
- [x] All plots are contour plots with outliers or pseudocolor plots.
- [x] A numerical value for number of cells or percentage (with statistics) is provided.

Methodology

Sample preparation	- Murine basal cell isolation : Tracheal tissues were cut into pieces and digested with 1.5 mg/ml Pronase (Roche, 10165921001) in DMEM/F12 medium (ThermoFisher Scientific, 31331-028) containing 100 units/ml Penicillin and 100 µg/ml Streptomycin (ThermoFisher Scientific, 15140122) at 4°C for overnight. After digestion, tracheal epithelial cells were pelleted, rinsed and resuspended in 8 ml Mouse Tracheal Epithelial Cells (MTEC) medium containing 10% FBS. Primary tracheal cells were expanded in MTEC proliferation medium containing 100 unit/ml penicillin and 100 µg/ml streptomycin. Cells were grown at 37°C in a 5% CO2 incubator, and medium was changed every other day. Single cells were obtained by incubation with 0.1% trypsin (ThermoFisher, 25300054) at 37°C for 20 min. Cells were counted, and for the staining 1×10.6 cells were used. Cells were labeled with 18 µg/ml rabbit anti-NGFR (Ab8875, Abcam) or IgG isotype control (Ab37415, Abcam) for 45min in 2% FBS, 2% BSA in PBS on ice. After incubation, cells were washed twice in 2% FBS, 2% BSA in PBS and incubated with Alexa Fluor 488 (ab150077, Abcam) diluted at 1:1000 in 2% FBS, 2% BSA in PBS for 45 min on ice.<br>- Murine inflammatory cell preparation : Inflammatory cells from BAL were centrifuged. Inflammatory cells from lung tissu were obtained after Percoll isolation method. Cells were counted by Turks staining and then incubated with an appropriate panel of antibodies (Supplementary Table 2) (BD, Franklin Lakes, NJ, USA) for 30 min in PBS 2% FCS before FACS analysis.
Instrument	BD LSR Fortessa cytometer (BD Bioscience) or FACSAria III (BD Bioscience)
Software	FlowJo v7.6.5 or FACSDiva softwares (BD Bioscience) (see Online methods /Flow cytometry or /Cytokine quantification or / FACS sorting procedure)
Cell population abundance	- Murine basal cell isolation : To determine the purity, we analysed the expression of cytokeratin 5 (CK5), which is a cytoplasmic protein expressed exclusively in basal cells from trachea, by indirect immunofluorescence. For this analysis, sorted cells were plated on a glass slide and fixed in acetone at -20°C for 10 min, washed 3 times in PBS and blocked with 3% BSA in PBS for 2 h at room temperature. Then, cells were incubated overnight at 4°C with rabbit anti-CK5 primary antibody (Ab53121, Abcam, 5µg/ml). After primary antibody incubation, cells were washed 3 times in PBS and incubated in the dark for 1h at room temperature with donkey anti-rabbit secondary antibody conjugated to Alexa 488 (R37118, ThermoFischer Scientific, 1/200 dilution). Cell nuclei were stained with DAPI. After washing 3 times in PBS, the glass slide was covered with Vectashield Mounting medium containing DAPI (H-1200, Vector Laboratories) and analysed by fluorescence microscopy (Zeiss Apotome). The method for assessing the purity was based on the counting of CK5+ cells. Number of total and CK5+ cells was counted manually throughout the glass slide using a 20x objective. Cell counting was performed on the basis of nuclear staining with DAPI and cell marker (CK5). After sorting, the airway basal cell purity was > 98%.
Gating strategy	On FACSDiva software, after adjusting our voltage and compensation settings (by using unstained and mono-stained control cells), we gated out debris (low left events on FSC vs SSC graph), cell doublets (on SSC-H vs SSC-W graph, and then on FSC-H vs FSC-W graph). To discern positive from negative cells, we used a non-immune Rabbit polyclonal antibody (IgG isotype, Ab37415, Abcam) to gated out unstained cells (see Supplementary Figure 12).

- [x] Tick this box to confirm that a figure exemplifying the gating strategy is provided in the Supplementary Information.
